# Integrating Quantum Mechanics into Protein-Ligand Docking: Toward Higher Accuracy and Reliability

**DOI:** 10.21203/rs.3.rs-5433993/v1

**Published:** 2024-12-05

**Authors:** Laszlo Fusti-Molnar

**Affiliations:** QuantumFuture Scientific Buda, TX, USA

## Abstract

I introduce two new methods, QFVina and QFVinardo, for protein-ligand docking that leverage precomputed high-quality conformational libraries with QM-optimized geometries and *ab initio* DFT-D4-based conformational rankings and strain energies. These methods provide greater accuracy in docking-based virtual screening by addressing the inaccuracies in intramolecular relative energies of conformations, a critical component often misrepresented in flexible ligand docking calculations.

I demonstrate that numerous force field-based methods widely used today exhibit substantial errors in conformational relative energies, and that it is unrealistic to expect better accuracy from the faster scoring functions typically employed in docking. Consistent with these findings, I show that traditional flexible ligand docking often produces geometries with significant strain energies and large deviations, with magnitudes comparable to the protein-ligand binding energies themselves and much larger than the differences we aim to estimate in docking hitlists.

By using physically realistic ligand conformations with accurate strain energies in the scoring function, QFVina and QFVinardo produce markedly different docking results, even with the same docking parameters and scoring functions for protein-ligand interaction energies. I analyzed these differences in docking hitlists and selected protein-ligand interactions using three protein targets from COVID-19 research.

## Introduction

Molecular docking is a crucial technique in structure-based drug discovery, providing insights into the binding modes and affinities of ligands when they interact with proteins. This computational approach predicts the preferred relative orientation of a ligand to the protein and identifies energetically favorable molecular conformations of the ligand within the active site of a protein. These predictions are essential for estimating binding affinity, a critical factor in drug design and optimization. Molecular docking generally involves two critical components: conformational sampling, where possible binding poses of the ligand are generated, and scoring, where these poses are evaluated based on their predicted binding affinities [1]. The success of docking studies depends significantly on the accuracy of both these steps.

Several prominent docking programs were developed early in this field. DOCK, the original automated docking program, introduced the concept of matching ligand spheres to receptor binding sites and used grid-based scoring functions [2]. Glide, a commercial software developed by Schrödinger, combines empirical and physics-based scoring components [3]. The Molecular Operating Environment (MOE) is another widely used platform, integrating docking with a broad array of computational chemistry tools. MOE’s docking module utilizes a force-field-based scoring function [4]. FlexX and Surflex-Dock are two other key programs from the early stage of docking development. FlexX uses an incremental construction algorithm that docks ligands by assembling fragments in the binding site, making it efficient for flexible ligands [5]. Surflex-Dock, with its empirical scoring function, is noted for its speed and user-friendliness [6]. The field of molecular docking and the estimation of protein-ligand binding energies has advanced rapidly over the last 10–15 years, driven by developments in machine learning and artificial intelligence. Alongside empirical and physics-based scoring functions, machine learning-based scoring functions have emerged as a third category. This has led to the development of new scoring functions, program packages, and numerous benchmark studies. Although these advances play important roles in current research, they are not directly relevant to the present work, so only a few representative examples are cited here [7–17].

Among the growing number of molecular docking programs, AutoDock Vina remains one of the most widely used due to its efficiency and accuracy. It introduced a scoring function in 2010 that combines empirical and knowledge-based approaches, significantly improving both the speed and accuracy of docking predictions compared to its predecessor, AutoDock [18]. Recent updates, including the development of the Vinardo scoring function, have further enhanced its capabilities by refining intermolecular interaction terms, particularly for hydrogen bonds and van der Waals interactions [19]. These advancements make AutoDock Vina and Vinardo particularly effective in a wide range of drug discovery projects, and numerous derivative program packages have already been developed based on AutoDock Vina. Sarkar et al. have published a comprehensive summary of these different Vina branches [20].

In this work, I propose a new docking approach that can be considered a new branch of the AutoDock Vina-based methods by combining Vina and Vinardo docking scores with quantum chemistry-based quantities. This approach decouples the tasks of finding conformer geometries, determining conformation order, and predicting strain energies from the docking process. This is achieved by using precomputed accurate quantum mechanical (QM) conformations with their respective orders and strain energies, based on high-level DFT-D4 calculations. The implementation of this method is straightforward and can be incorporated into any well-designed docking program. I have implemented this new docking scheme using the current version of the AutoDock Vina program, without modifying the docking package itself. I call these two new schemes QFVina and QFVinardo, indicating that QuantumFuture’s QM conformational database is used in combination with Vina and Vinardo partial docking scores. The scheme is simple: instead of traditional flexible ligand docking, where the program must find all feasible conformations and their internal energies, we perform rigid ligand docking using precomputed low-energy QM conformations. The docking program’s scoring function provides the intermolecular interaction energy and desolvation energy, implicitly parameterized in the empirical scoring function in AutoDock Vina. The intramolecular energy contribution of the ligand comes from the precomputed DFT-D4 conformational strain energies. The sum of these values forms a new scoring function for estimating the binding energy between the protein and the ligand. Additionally, precomputed QM quantities, including thermodynamic properties of each conformation and the total conformational Gibbs free energy of the ligand, can further refine hit lists and provide an initial approximation of the binding free energy in protein-ligand interactions.

The main motivation for this work will be demonstrated in the next section by analyzing and extending an existing database for studying conformational relative energies and rankings of drug-like molecules using widely adopted methods. It will be shown that the challenges in predicting conformational relative energies are often underestimated by the docking community. Current force fields, semi-empirical QM methods, and advanced AI-based methods, such as ANI family of methods, exhibit deficiencies in accuracy compared to state-of-the-art *ab initio* methods. The scoring function in docking, on the other hand, needs to be significantly faster than even the fastest force fields analyzed in the next section. Thus, relying on such scoring functions to identify physically realistic conformational geometries and to estimate rankings and relative energies of conformations may be irrational, regardless of whether the scoring function is empirical, physics-based, or machine learning-driven.

I will provide details on our current QM-based conformational libraries, designed for drug repurposing research, in the following section. Practical development aspects of QFVina and QFVinardo docking will be presented afterward followed by discussions about computational costs. Results will then be compared to the traditional flexible ligand Vina and Vinardo docking outcomes by analyzing strain energies and docking hit lists, using three targets from COVID-19 research as examples. Finally, conclusions and potential next steps for further development will be discussed in the last section.

## Benchmarking the Conformational Strain Energies and Rankings

Folmsbee and Hutchison published a significant study [21] investigating conformational energies using various computational methods, including *ab initio*, semi-empirical QM, force field, and machine learning methods such as the ANI family. They collected up to 10 conformations for approximately 700 molecules, resulting in over 6,500 molecular geometries. These geometries were obtained via dispersion-corrected DFT-D3 geometry optimizations, with high-quality DLPNO-CCSD(T) triple-zeta energies calculated and published as reference energies. I downloaded their geometries and results from their GitHub site and extended the study with the following additions:

1. I added new DFT results, partly to validate our new DFT-D4 program against this dataset, ensuring it provides consistent results with the published values and confirming the accuracy of all calculations. Additionally, I extended the range of the DFT functional/basis set combinations presented.
2. Additional force fields were included, and some force field calculations were repeated to address inconsistencies in the original results.
3. As a quality measure, I calculated the means and medians of the RMSD of the relative energies of the conformations for the same molecules. I also calculated the means and medians of the ranking errors, defined by how many conformations, using the given method, had lower energies than the lowest energy conformation determined by the reference DLPNO-CCSD(T) method.
4. I present two sets of statistics, ensuring that no data is missing. All methods listed in both tables below have energies for all geometries considered in the statistics. Two separate sets of statistics were necessary because the ANI family of methods only supports about half of the results due to the limit of supported elements.

Tables 1 and 2 below present statistics for 2,954 and 6,612 geometries, respectively, with Table 1 including the ANI family of methods.

The statistics in Table 1 and Table 2 are quite similar for methods represented in both, indicating that the statistics in Table 1, which use less than half of the dataset, are realistic. The DFT methods, almost regardless of the basis set and functionals used, distinguish themselves by having mean RMSD values of relative energies below 0.5 kcal/mol, with some below 0.4 kcal/mol, and mean ranking errors around or mostly below 1.0. The second category of quality appears to be the semi-empirical QM methods and the ANI family of methods, with approximately double the error in both the mean RMSD of relative energies and the mean ranking errors compared to DFT methods. Interestingly, even the ani1cc method, developed to surpass dispersion-corrected DFT accuracy, performs similarly to semi-empirical methods and falls short of DFT accuracy. With computational costs similar to semi-empirical calculations and support for only a limited number of elements, the ani1cc method currently seems to be an inferior choice compared to existing semi-empirical methods.

The third category of quality includes force field-based methods, with the GFN-FF method being an exception, showing performance close to the semi-empirical QM level. This analysis highlights that widely used force fields generally lack the accuracy needed to estimate conformational relative energies and ranks. Even semi-empirical QM methods and the most advanced AI-based models, which have computational costs similar to QM semi-empirical methods, fall short in accurately predicting conformational strain energy and ranking. The physics-based, empirical, or AI-based scoring functions used in current docking programs are even less accurate than the force fields mentioned above, as they require lower computational costs. Due to this inaccuracy in conformational strain energies, docking hit lists are often filled with false positives. Even if docking results identify favorable conformations with strong protein interactions, the scoring functions cannot accurately determine whether the conformation has zero or 5 kcal/mol strain energy, or anything in between. Consequently, hit lists must be larger than desired to ensure true positives are identified, as the number of false positives is large and increases with the size of the docking project.

## Introducing QM Based Conformational Libraries for Drug Repurposing

In this section, I will present details of two such precomputed QM databases. One is based on FDA-approved drugs and includes nearly 1,600 drugs. The other is derived from the COCONUT natural compounds database[26, 27], currently covering approximately 4,000 natural compounds. These databases are designed for drug repurposing research and are incomplete at this time. As computational resources expand, I plan to include the remaining FDA-approved drugs and extend the database to cover most approved drugs worldwide. I also plan to expand the COCONUT QM library, as the COCONUT database itself contains hundreds of thousands of natural molecules. The summary of the QM conformational data structure can be conveniently displayed using the tree command in Linux with the --filelimit option, which shows the number of files in each directory. The summary for the FDA-approved drug database is shown below, followed by a more detailed explanation.

The construction details of the two databases are as follows:

1. *Input Data Preparation Steps with Python Script Using RDKit*
  I downloaded 2,083 starting structures from the MOLDB website and applied the Weber filter, which restricts the number of rotatable bonds to a maximum of 10 and limits the topological polar surface area to below 140 Å^2^. This filtering reduced the number of drug molecules to 1,642. I ran our qfLowerLevel.x application to optimize the geometries, ensuring valid quantities in all SDF files (though this step was not strictly necessary for the subsequent steps). I filtered out one additional structure due to an SDF issue, resulting in 1,641 drug molecules as the starting inputs for this project.
  For selecting natural compounds, I began with the COCONUT database, which contains over 400,000 natural compounds (as of January 2024). I selected about 1% of the compounds for this database by applying the Lipinski, Ghose, Veber, Reos, and drug-like filters, and limiting the number of atoms to the range of 28 to 62. This selection resulted in 3,987 compounds, with 3,985 having acceptable SDF files for our current SDF reader.
2. *Finding the Conformations with the qfconfsearchDFT.x Application*
  I ran our qfconfsearchDFT.x application with default parameters, utilizing the revSCAN functional with the new D4 dispersion correction parameters and the def2-TZVP basis set in all DFT-D4 calculations. This functional/basis set combination has proven to be highly accurate in both our intermolecular interaction benchmarks using the S66×8 standard set and our study comparing calculated vacuum conformations to high-quality experimental geometries [22]. I selected 1,600 successful results in vacuum and 1,610 in aqueous continuum for FDA-approved drugs, and 3,892 and 3,917 for natural compounds in vacuum and aqueous continuum, respectively. A small number of calculations showed unwanted behaviors, where the original bonds from the SDF file were altered during QM semi-empirical geometry optimizations. We excluded these cases since we are focusing on conformations of the same molecules in this project, not investigating tautomers or chemical reactions.
  The QFConfs_ALL directories contain all the results. Each SDF file includes all important conformations found with our application, along with the DFT-D4 (revSCAN, def2-TZVP) energies and the relative energies compared to the lowest energy conformation (conformational strain energies), attached as standard SDF tags. Note that the numbering in the file names has no significance; it simply reflects the order of files during calculations. To locate the SDF file for a given drug or natural compound, one can use grep with the corresponding name or CAS number or COCONUT’s ID.
3. *Further Deduplication of the Conformations Using the qfdeduplicate.x Application*
  I introduced two new directories: QFConfs_DiversityLevel0 and QFConfs_DiversityLevel1. Data files in these directories require some explanation. The qfconfsearchDFT.x application uses RMSD pairwise alignment-based deduplication via the C++ APIs of RDKit, by default, only for molecules with no more than 40 atoms. We include hydrogen atoms in RMSD alignments and deduplication because we aim to retain important conformations with different hydrogen orientations. However, RMSD calculations are computationally expensive and scale poorly with molecular size. Some tests showed that RMSD-based deduplication could be more expensive than all ab initio DFT-D4 calculations. Therefore, we limited RMSD-based deduplication to molecules with up to 40 atoms and developed an alternative scheme for larger molecules. This alternative scheme is new and experimental, currently set conservatively, meaning it sometimes retains structures that are very similar. This conservative approach is preferable, as eliminating duplicates later is easier than recovering missing conformations, which would require repeating expensive calculations.
  The QF_DiversityLevel0 and QF_DiversityLevel1 directories contain post-processed deduplicated conformations. We used a 0.3 Å RMSD limit in the QF_DiversityLevel0 directory and a 0.6 Å RMSD limit in the QF_DiversityLevel1 directory. Note that if the energies of two conformations differ by more than 0.3 kcal/mol, we keep both, regardless of the RMSD difference in geometries. Further deduplication or different deduplication starting from the conformations in the QFConfs_ALL directories is possible, and users can easily perform this themselves. Unfortunately, one structure failed to process, leaving us with 1,599 instead of 1,600 structures in vacuum and 1,609 instead of 1,610 in aqueous continuum for the FDA-approved drugs database.
4. *Going Beyond Ab Initio DFT-D4 Energies and Strain Energies*
  In addition to accurate ab initio DFT-D4 energy orders and strain energies of conformations, this database provides extra quantum mechanical information. I performed statistical thermodynamic calculations using the GFN-XTB and PM6-D3H4X quantum mechanical semi-empirical methods for all conformations of all drug molecules with our qfLowerLevel.x application. With all necessary thermochemistry quantities for all conformations, I also calculated the Gibbs free energies for the conformational ensemble of all drug molecules using our qfensamble.x program. The Gibbs free energies for the conformational ensembles are written to individual log files for each SDF file by adding a simple .log extension, as this value pertains to the entire conformational ensemble of the given drug molecules. As a result, the number of files is doubled in all directories of thermochemistry calculations.

## Discussion about computational costs

The computational expense of QFVina and QFVinardo is greater than that of traditional flexible ligand Vina and Vinardo docking, but not prohibitively so. QFVina and QFVinardo perform docking by looping over all stored conformations of a given ligand. For flexible ligands, the number of conformations is typically a few dozen, and we retained up to 100 conformations during the construction of the QM conformational databases. In contrast, traditional Vina and Vinardo perform a single flexible docking procedure with the same ligand, but flexible docking is inherently slower than the rigid body docking used by QFVina and QFVinardo.

Overall, the slowdown due to looping over all conformations and performing rigid body docking is about 5- to 10-fold on average, depending on the number of conformations used for the ligands. Additionally, it is possible to speed up QFVina and QFVinardo by creating a derivative QM conformation dataset through further deduplication using higher RMSD thresholds. This process results in a more diverse but reduced set of conformations for each ligand, thereby reducing computational demand.

The more significant computational expense is not the docking itself, but rather the creation of the QM-based conformational database. Specifically, this involves performing *ab initio* DFT calculations with a high-quality functional and basis set for every conformation of every ligand. These calculations are essential for obtaining the final accurate orders of the conformations along with their precise DFT-D4 total energies and strain energies. One of the flagship products of my company, QuantumFuture Scientific, is an extremely fast DFT program that I developed over the last few years. This new program builds on methodological advancements made about two decades ago [28–33]; however, with two decades of additional experience and insight, both the quality and performance of this new DFT code are several classes above the developmental code we were working on in an academic environment back then. This new DFT program is used for this task within the QFConfsearchDFT application. The details of all QF applications are described in [22], and I include two relevant figures below that illustrate computational speed benchmarks of DFT calculations compared to other widely used popular ab initio programs.

**Fig3:**
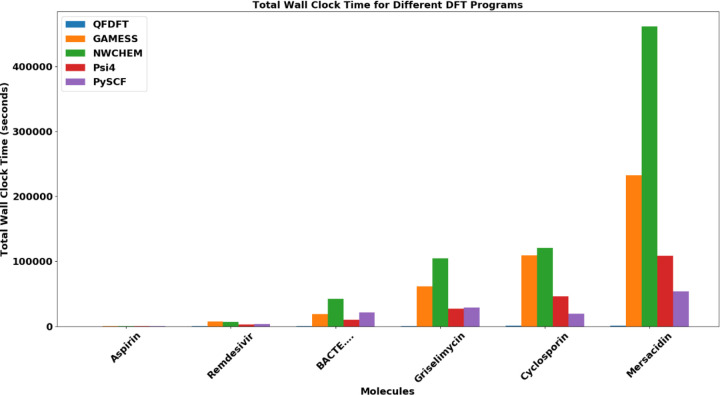
DFT benchmark calculations with different programs for a series of increasingly larger drug molecules using def2-SVPD basis set and TPSS functional on an Intel I9 18 cores workstation

**Fig4:**
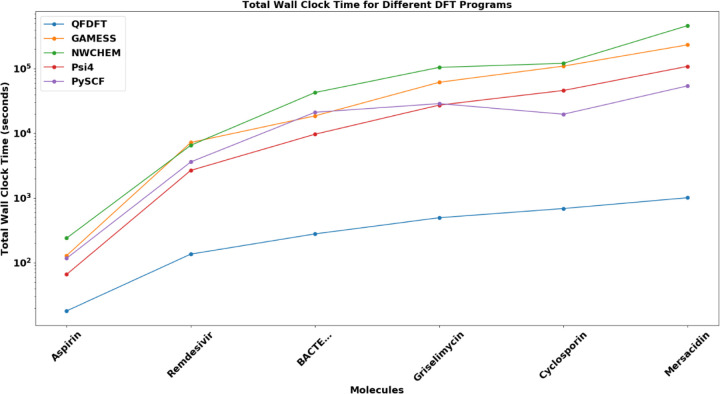
DFT benchmark calculations with different programs for a series of increasingly larger drug molecules using def2-SVPD basis set and TPSS functional on an Intel I9 18 cores workstation. The calculation costs are in logarithmic scale

Our DFT program provides the same or better accuracy than the other programs listed above (see the details in [22]). It is so much faster that a logarithmic scale was necessary in Figure 4, as its computational costs, compared to the other programs, are almost completely invisible on Figure 3.

Building the two QM-based conformational databases introduced above was relatively straightforward, especially with our new implementation for Azure. The overall computational cost with our DFT program was moderate, and with a more significant one-time investment, it would be possible to build much larger QM-based conformational databases, orders of magnitude larger, for general-purpose drug design. This brings us to a broader discussion on computational expenses, which is increasingly important as the world faces growing challenges related to energy usage.

It’s crucial to distinguish between local computational expenses and global expenses. Traditional docking programs typically generate ligand conformations on-the-fly from input structures, often using SMILES strings or 2D SDF files sourced from general databases. These conformations are generated quickly, with small local costs, either by the docking programs themselves or by third-party software. However, even within a single research group, the same conformations may be generated multiple times over weeks, months, or years. When considering how many research groups around the world do the same, it’s likely that the same (low-quality) conformations are being generated hundreds of thousands, if not millions, of times independently each year.

While each local cost is small, the cumulative global cost in terms of CPU (or GPU) usage and energy consumption is potentially very large. This cumulative expense could be comparable to the cost of creating a high-quality QM conformational database once and for all. Rather than having every researcher independently generate low-quality conformations, everyone could rely on a shared, accurate QM database. This approach would not only optimize computational and energy resources on a global scale but also provide much greater accuracy in docking applications.

## Development details for QFVina and QFVinardo Docking Methods

As mentioned above the concept behind the QFVina and QFVinardo docking schemes is to decouple the complex tasks of finding physically feasible low-energy conformations and accurately estimating conformational ranks and strain energies from the docking process. These tasks are handled separately using more reliable QM calculations, which have a better chance of achieving these goals accurately. Once such QM-based conformation libraries are prepared, the AutoDock Vina program can be used for docking by considering all conformations of all ligands in the given QM conformational database as rigid ligands, serving as an alternative to flexible ligand docking. The protein target can be either rigid or flexible, as in traditional AutoDock Vina docking; however, this first version of the software focuses on rigid protein docking.

To facilitate large-scale docking calculations, my software development centered on three main components:

1. *Developing a New Application:* This application (qfvina.x) performs the necessary ligand preparations, conducts the docking, and generates the docking results as standard SDF files.
2. *Creating a Docker Container:* This container installs all necessary third-party libraries and programs, as well as my main docking application and a few utility applications.
3. *Automating the Docking Process on Azure:* This application launches an entire docking project on Azure automatically, using only a few command-line options. It downloads the Docker container, starts the specified virtual cluster (exclusively using inexpensive spot instances), performs all calculations, shuts down the cluster after completion, and stores the results on Azure Blob Storage until they are downloaded and deleted from temporary cloud storage by the user.

After completing these three steps, a simple command such as:


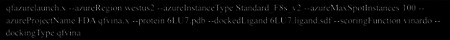


will launch the docking calculations on Azure in the westus2 region, using one hundred 8-vCPU Xeon spot instances. The QM conformational library is in the FDA.tar.gz file in this case, as defined by the value of the --azureProjectName argument. The command tells the qfazurelaunch.x application to use the qfvina.x scientific application on Azure, with the rest of the command-line arguments pertaining to that specific application. A simple GUI has been developed, as well, for launching calculations like this on Azure for users who prefer to avoid commands like above. This simple GUI provides the way to accomplish the same task with only a few clicks of the mouse.

The qfvina.x application, described in the first component above, accepts several command-line arguments, including --dockingType, which can be set to qfvina for the new QFVina or QFVinardo methods, or vina for traditional flexible ligand docking. The --scoringFunction argument accepts either vina or vinardo options. The --protein argument specifies the target PDB file, and an optional --ligand argument can define the ligand SDF file. If not specified, the scheme to perform all docking automatically handles it internally. The --dockedLigand from the downloaded PDB structure is used only to define the center of the docking box as the geometric center of the docked ligand. If not specified, the --centerX, --centerY, and --centerZ arguments must be used to specify the center of the docking box. In this article, we used the docked ligands from the original PDB file to define the docking box center. There is also a --boxsize argument that defines a cubic docking box, and we may introduce separate inputs for X, Y, and Z directions in the next version. For all docking calculations in this work, we used a 20 Å × 20 Å × 20 Å cubic docking box.

The qfvina.x application calls the third-party program mk_prepare_ligand.py from Meeko [34] for ligand preparation and to obtain the input ligand in the necessary PDBQT format for AutoDock Vina docking, as well as the mk_export.py program to export the results to standard SDF format. It also calls wk_prepare_receptor.py from the Waterkit program cite [35, 36] followed by mk_prepare_receptor.py [34] for the necessary protein preparation for AutoDock Vina. The exhaustiveness parameter for AutoDock Vina was set to 32 for all docking runs. We kept the top 5 docking poses for all conformations in QFVina and QFVinardo runs, and the top 50 docking poses for traditional Vina and Vinardo flexible ligand docking. Although this is likely an overkill and the storage space for results could be reduced by decreasing these values in future projects, I retained these values for safety.

## Docking Results and Discussion

In this section, I present docking results using our new QFVina and QFVinardo docking schemes, comparing them with traditional flexible ligand docking using Vina and Vinardo scoring functions. We selected COVID-19 targets for this analysis, although this study is not intended for true drug repurposing at this stage. The focus is on a detailed comparison of the results for three protein targets, specifically analyzing the differences between traditional docking methods and QM conformation-based docking.

I will demonstrate that the hitlist produced by QFVina is significantly different from the one produced by Vina, and similarly, the hitlist generated by QFVinardo is markedly different from that of Vinardo. This observation alone supports the validity of the QFVina and QFVinardo methods, as QM conformations are much more realistic than those generated by flexible ligand docking programs. If these differences in conformations lead to significant variations in the final hitlists, it suggests that QM conformation-based docking is also likely to be more accurate in most cases.

At the beginning of this section, I analyzed the differences in detail between using accurate QM-based conformations and *ab initio* DFT-D4 conformational strain energies in docking versus the traditional docking scheme. I downloaded two protein structures for the COVID-19 main protease (PDB ID: 6LU7 [37] and PDB ID: 6Y2G [38]) and a protein structure for the COVID-19 spike protein (with the PDB ID:7BZ5 [39]) from the PDB database. Docking was performed using all conformations from our FDA-approved drug conformation database and our natural compounds conformation database, applying the QF approach with both Vina and Vinardo scoring functions for all three targets. To enable comparisons, traditional flexible ligand docking was also performed for the FDA database using both Vina and Vinardo scoring functions for these three targets.

For each conformation of each molecule, the QFVina method retained the five best-scored docking solutions, while the traditional Vina method retained up to the 50 best-scored docking solutions. After completing all docking calculations, our analysis program collected the results, sorted them by score, and retained the top 100 solutions for each method, target, and scoring function combination. This top 100 hitlist represents the lowest binding energies of all conformations and poses, with the same molecule potentially appearing multiple times. To address this, I also created a condensed hitlist, where each molecule is represented only once by its strongest binding entry from the top 100 hitlist. The two tables below present the binding energy intervals for all our docking results.

One observation is that the Vinardo scoring function generally estimates slightly weaker protein-ligand binding energies, often by a few kcal/mol. This trend is consistent for QFVinardo compared to QFVina, suggesting that the difference arises from variations in the intermolecular and desolvation energy scoring functions, since the intramolecular energy contributions come from DFT, which we will analyze in detail below.

The most critical analysis for this article focuses on the conformational strain energies in all our results, as the primary objective of the QFVina method is to avoid solutions involving physically non-existent conformations with unreasonably high strain energies. The strain energies for all QFVina docking results can be easily obtained by subtracting Vina energy from the DFT+Vina energy in the final SDF files, as both quantities are included as standard SD tags by our software. Since we perform docking with rigid ligands, the DFT energies remain valid, and the conformational geometries stay in their QM local minimum. The figure below displays the conformational strain energies for both Vina and Vinardo scoring functions across all three targets, while the table below presents key basic statistics of the conformational strain energies.

As observed, many docking solutions in the top 100 lists correspond to the lowest DFT strain energy conformations, and when this is not the case, they are based on conformations with very small strain energies. This outcome is expected, as the thermal energy k_B_T at room temperature is about 0.6 kcal/mol. Although very favorable intermolecular interactions between the protein and ligand can sometimes overcome much larger conformational strain energies, similar to intermolecular interactions in organic crystals, it becomes increasingly difficult for conformations with higher strains not only from thermochemistry but also from reaction kinetics point of view as well. These results clearly show that the docking program identified very reasonable poses with strong intermolecular interactions with the protein while maintaining low ligand strain energies, with all means and medians of the DFT strain energies below 0.5 kcal/mol and very small deviations.

When comparing the Vina and Vinardo scoring functions, Vinardo consistently favors conformations with even smaller DFT conformational strains than Vina. This effect, although small, is consistent and can be explained by our earlier observation that Vinardo generally estimates weaker intermolecular energies than Vina. Weaker intermolecular interactions can compensate less for intramolecular strains. Despite these small differences, it might be beneficial in practice to perform both QFVina and QFVinardo docking and combine the results.

For the traditional Vina docking solutions, some additional calculations are required. First, hydrogens need to be added to the docking poses, which can be easily done using standard cheminformatics tools. We accomplished this using RDKit Python APIs. Second, geometry optimizations with constraints are necessary, where all hydrogen positions are optimized while keeping all heavy atom coordinates fixed. This can be easily achieved using our QF applications, as both the QFDFT and QFLowerLevel applications include a dedicated Boolean keyword in the geometry optimization module for this specific task. To ensure the most consistent comparisons, it is also important to use the same methods here as were used when constructing the conformational databases with the QFConfSearchDFT program. This program utilizes the GFN-XTB semi-empirical QM method for geometry optimizations of all final conformations and calculated strain energies using DFT with the revSCAN functional and def2-TZVP basis set, both of which are default options in the QFDFT application.

Therefore, if all (in this case, the top 100 hits) SDF files from the docking results are in the current directory, followed by hydrogen additions with RDKit, a simple shell script like the following can be used for both calculation steps:


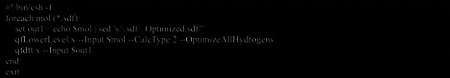


The DFT conformational strain energies can be obtained by subtracting the DFT energy from the qfdft.x output for all docking structures from the lowest energy of the same drug molecule in our conformational database. The table below presents the same statistical measures for strain energies using Vina and Vinardo scoring functions in flexible ligand docking, while the figure below illustrates the strain energies for all methods and targets.

There is a small negative number in the table above, with a value of −0.12 kcal/mol, which requires some explanation. In this case, Vina identified the same conformation as the lowest-energy conformation in our database. After optimizing the hydrogen atoms, this conformation resulted in a slightly lower energy structure in terms of DFT-D4 energy than the one stored in our database. While this may appear unusual, it is not entirely unexpected, as all final conformations in the database were optimized using the GFN-XTB QM semi-empirical method. Naturally, it is possible to find molecular geometries with even lower DFT-D4 energies. Considering the minimum values in the table, it is important to note that this is the only conformation, out of all 6×100 poses in the hitlists analyzed above, where either Vina or Vinardo found a conformation close to the lowest-energy conformation in our QM conformation database.

As demonstrated by the statistics in the table and shown also on the figure above, the strain energies from the docking solutions are extraordinarily high, even after properly adding all hydrogens and optimizing their geometry using the same QM method employed in constructing our FDA conformational database. The problem lies not only in the extremely high strain energies but also in the large deviations in these strain energies, which are on the same order of magnitude as the protein-ligand binding energies we aim to estimate. The molecular structures of the top 100 hits from the flexible ligand docking results represent non-physical molecular geometries and conformations with very high internal strain energies.

These results raise the question of why the widely used docking solutions are considered useful at all and how they can yield meaningful results from such overwhelmingly non-physical solutions. The answer is that they can work, at least sometimes, and to some extent. Consider, for instance, rigid molecules with no rotatable bonds and only one conformation. In such cases, conformational strain energy plays no role; it isn’t even defined with only one conformation. If the protein-ligand binding free energy is reasonably estimated as the sum of the intermolecular interactions and desolvation energies in the scoring function, the docking program can identify important drugs correctly or at least quasi-correctly. The geometry of these important drugs may have high strain, but using the true realistic geometry, the interaction between the protein and ligand, as well as the desolvation energies, would be about the same. In other words, the protein-ligand binding free energy for the non-physical, highly strained geometry and the realistic geometry of the given molecule is roughly the same, making the choice of geometry less critical.

Another scenario involves a flexible molecule with many conformations, where several conformations interact strongly with the protein. Even if the docking scoring function cannot rank the conformations correctly, the docking project can still succeed if one conformation interacts strongly with the protein and has low internal energy according to the scoring function. However, this success might occur for the wrong reason, as the docking conformation may not be a low energy, physically occurring conformation. Instead, another truly low-energy, physically existing conformation at room temperature might interact similarly strongly with the protein. Despite the inaccurate rationale, the docking project identifies this drug molecule, and the project is considered successful.

The problem arises when only one or a small group of conformations show strong interactions with the protein, which is often the typical case. If the docking scoring functions inaccurately estimate higher intramolecular energies, these results will be ranked lower on the hit list, and the docking will miss that drug molecule, leading to a false negative. Similarly, when conformations that show strong interactions with the protein are deemed energetically feasible by the scoring function, but in reality, they are not due to large conformational strain energies that the scoring function fails to estimate correctly, these false positives fill the top of the hit list, pushing down the true positives. Unfortunately, these two issues tend to occur together. Introducing a relatively flat intramolecular model scoring function, where the conformational strain energies are smaller in magnitude than the protein-ligand intermolecular interactions, could reduce the number of false negatives but would simultaneously increase the number of false positives, leading to no overall improvement in outcome.

Since neither the Vina nor Vinardo scoring functions produce reliable low-energy structures or conformational rankings in flexible ligand docking, the outcome depends largely on luck, determining how many false positives and false negatives are present in the docking project. This is why I developed the QFVina docking scheme, where accurate and reliable DFT-D4 conformational strain energies are obtained from the input conformations. Combining this with the Vina intermolecular (and implicitly desolvation) scoring could significantly reduce both the number of false positives and false negatives, providing a more reliable docking hit list.

In the second half of this section, I will analyze our docking hit lists in more detail. Out of the 24 known active drugs identified in experimental work from [40], 14 FDA-approved drugs are included in our 1,599 conformational database. (This highlights that our database is incomplete and underscores the importance of expanding it, at least to near-completeness regarding FDA-approved drugs and potentially including additional approved drugs from the rest of the word.)

The 14 experimentally active drugs against COVID-19 in our database are: *LOPERAMIDE, MEFLOQUINE, M AMODIAQUINE, AMODIAQUINE, HEXACHLOROPHENE, HYDROXYPROGESTERONE CAPROATE, M CICLESONIDE, CICLESONIDE, GILTERITINIB, ABEMACICLIB, IVACAFTOR, BAZEDOXIFENE, NICLOSAMIDE*, and *ELTROMBOPAG*. It is possible that additional FDA-approved drugs active against COVID-19 exist if the experiments did not include all of them or if some experimental results were not entirely reliable.

Combining the QFVina and QFVinardo results, we identified five drug molecules from the above list of 14 by considering only two proteins for the main protease and one for the spike protein. NICLOSAMIDE ranked 18th on the top hit list for the 6LU7 target, with one of its low-energy conformations having 0.061 kcal/mol conformational strain energy and a total binding energy of −8.36 kcal/mol, which includes the Vina binding energy and the DFT conformational strain energy. ELTROMBOPAG was the 27th strongest binding drug for the 6LU7 target, with a binding energy of −8.197 kcal/mol, and it was the lowest energy conformation in this case. CICLESONIDE was the 35th strongest binding drug for the 6LU7 target, also with its lowest energy conformation, showing an estimated binding free energy of −8.109 kcal/mol.

QFVinardo docking also identified NICLOSAMIDE as the 4th strongest binding drug for the 6LU7 target, with the same low-energy conformation having 0.061 kcal/mol conformational strain energy and a total binding energy of −6.54 kcal/mol. Regarding the other target, QFVina identified GILTERITINIB as the 21st strongest binding drug for the 6Y2G target, with its lowest energy conformation and an overall binding energy of −9.997 kcal/mol. QFVina also identified ELTROMBOPAG as the 5th strongest binding drug for the 7BZ5 target, with its lowest energy conformation and an overall binding energy of −10.289 kcal/mol. QFVinardo further improved the ranking for ELTROMBOPAG, identifying it as the 2nd strongest binding drug for the 7BZ5 target, with an estimated binding energy of −7.955 kcal/mol. Finally, QFVinardo identified IVACAFTOR as the 36th strongest binding drug for the 6Y2G target.

Interestingly, traditional flexible Vina docking with Vina scoring functions and the same inputs, docking box size, etc., did not identify a single active drug from the list of 14 above. Using the Vinardo scoring function, the situation improved, identifying two known active drugs from the list of 14: NICLOSAMIDE, which ranked 24th for inhibiting the 6LU7 target, with an estimated binding energy of −5.981 kcal/mol, and ELTROMBOPAG, which inhibited all three targets.

In summary, using traditional flexible Vina docking combined with both Vina and Vinardo scoring functions, we identified two drugs experimentally verified to be active from our list of 14: NICLOSAMIDE and ELTROMBOPAG. They did not identify any other active drugs from the list of 14 that QFVina or QFVinardo did not find. QFVina and QFVinardo identified both of the above active drugs and an additional three drugs from the list of 14 missed by traditional flexible docking with Vina and Vinardo scoring: CICLESONIDE, GILTERITINIB, and IVACAFTOR. I emphasize that these few docking examples are merely an initial demonstration that QFVina and QFVinardo docking could yield reasonable results, potentially offering advantages over existing docking technologies. Much larger and more rigorous studies are needed to fully assess their true potential.

The figure below presents a condensed version of the top 100 list, where each drug molecule is listed only once, regardless of how many times it appears with different conformations and docking poses. It also shows the estimated binding energies with and without the DFT conformational strain energies for the QFVina and QFVinardo results, and the last column indicates how many conformations and docking poses each drug represents in the top 20 of the top 100 list.

After discussing the known active drugs, we identified for COVID-19, it is interesting to analyze some false positives. Sunitinib, the top molecule on the QFVina list, serves as an example for deeper analysis. It is important to note that more than 25 drugs are within 1 kcal/mol of each other in their estimated binding free energies, and the accuracy of the docking scoring function is not close to 1 kcal/mol. Therefore, all drugs on the list warrant further detailed analysis, and any of them could serve as an example. Sunitinib was chosen simply because it tops the list.

Interestingly, traditional flexible ligand docking with the Vina scoring function did not identify this drug in the top 100 list. However, it is ranked third on the QFVinardo list. The figure below, created using the third-party DiscoveryStudio program for visualization, highlights numerous attractive interactions between the drug molecule and the protein. There is one unfavorable donor-donor interaction that might be underestimated by both the Vina and Vinardo scoring functions, which could contribute to the falsely strong binding estimations. However, there are also several attractive hydrogen bonds and other favorable interactions, as the figure indicates.

Based solely on these docking results and visual inspection, it is impossible to definitively determine whether Sunitinib is a true false positive or to identify the exact cause if it is. This example serves as a reminder of the necessity for further investigation of all docking results using higher-level and more accurate absolute binding free energy methods, where more reliable quantitative binding free energies can be obtained.

It is worth noting that *Sunitinib* has been reported to be effective for COVID-19 treatment, although not necessarily through inhibition of the main protease. For more information, see the statement “suggesting sunitinib is likely to be an inhibitor for dangerous coronaviruses infection with broad spectrum” from the paper [41].

The top-ranked molecule from the QFVinardo docking results is *Phenazopyridine*, although approximately a dozen other drugs are within 1 kcal/mol of its estimated binding free energy at the top of the QFVinardo list. This case is particularly interesting because QFVina, using the same QM conformations, did not identify *Phenazopyridine* within its top 100 hits. This discrepancy highlights some differences in the intermolecular interaction assessments between the QFVina and QFVinardo scoring functions. It also illustrates that the field is highly competitive, where even small differences in scoring functions can completely remove top-ranked lead candidates from the hit list. This underscores the importance of every bit of accuracy improvement in docking, as even minor enhancements could lead to very significant improvements in the results.

**Fig9:**
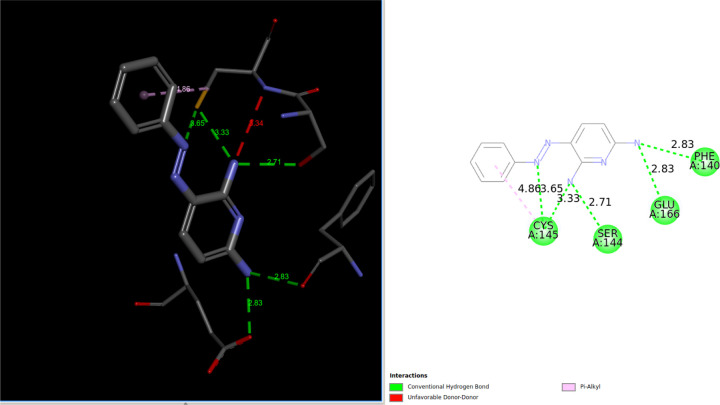
Intermolecular interactions between the lowest energy QM conformation of Phenazopyridine and the 6LU7 protein target

*Phenazopyridine* is a relatively small and rigid drug molecule. Its lowest energy QM conformation is approximately 2.2 kcal/mol lower in DFT-D4 energy compared to its second-lowest energy conformation. As illustrated in the figure 9 below, this conformation forms several hydrogen bonds with surrounding residues, suggesting potential activity against the target protein. However, there is also an unfavorable interaction between two N-H groups that might be underestimated by the Vinardo scoring function, similar to the observation made with *Sunitinib*. This underestimation could contribute to an overestimation of the binding affinity, potentially classifying Phenazopyridine as a false positive in this context. Nevertheless, this remains speculative without further detailed analysis.

Importantly, Phenazopyridine has been reported to exhibit activity against COVID-19, supporting the possibility that it is not a false positive in our docking studies. For more detailed information, refer to the experimental findings reported in [42].

Similar condensed versions of the top 100 hit lists are shown in the two figures below for the two other targets. An interesting example from the top sections of all four methods is M Midostaurin. This case illustrates the scenario discussed earlier, where a drug molecule exhibits such strong intermolecular interactions with the protein that not just one, but many different poses and conformations interact strongly with the protein.

*M Midostaurin* dominates the top 20 list in both the QFVina and traditional flexible ligand docking hit lists with Vina scoring for the 6Y2G target, occupying 13 out of the top 20 spots. It also leads the QFVinardo list, taking 10 of the top 20 positions, and appears twice in the top 20 list of the traditional flexible ligand docking with Vinardo scoring. Additionally, it appears on the hit list of the 7BZ5 target, as well.

These outcomes either suggest strong inhibition potential for the spike protein or indicate a potential overestimation of one or more specific intermolecular interactions by the scoring functions. More accurate and higher-level binding free energy calculations could provide further insights, particularly if such calculations are applied across the entire top 100 list of drug molecules.

Since *M Midostaurin* appears to be a very potent inhibitor based on the docking results, I examined the molecule more closely. There are three separate M Midostaurin molecules in the current QF FDA-approved database, which consists of 1,599 drugs. Two of them are 3-Hydroxy Midostaurin, and they not only share the same name but also the same CAS number, 179237–49-1. These molecules are stereoisomers, as illustrated in the picture below.

The S stereoisomer of *3-Hydroxy Midostaurin* is more active because its OH group is oriented toward the GLU166 residue, allowing it to form an additional strong hydrogen bond. This S stereoisomer consistently appears at the top of the hitlists for both QFVina and QFVinardo. The interaction between this stereoisomer and the 6Y2G target protein, as determined by the QFVinardo method, is illustrated below in both 2D and 3D visualizations. These images highlight all the important intermolecular interactions between the ligand and the protein. Notably, Midostaurin has been suggested for drug repurposing to treat COVID-19 patients [37]. These docking results strongly indicate that the S stereoisomer of the 3-Hydroxy Midostaurin may effectively inhibit the COVID-19 protein, and I recommend further experimental testing to explore this potential.

Next, a brief analysis of the QFVina and QFVinardo docking results is presented using our library of nearly 4,000 conformations of natural molecules from the COCONUT database [26]. The COCONUT ID is provided for identification, and this ID can be used to access numerous physical and chemical statistics of the molecules on the COCONUT website, as well as different standard and alternative names that can be used for toxicity prediction on other third-party web services.

Regarding drug repurposing, natural products offer an interesting avenue since their number is about two orders of magnitude greater than the number of FDA-approved molecules (although our current QM conformational library contains only about 4,000 molecules). Additionally, the probability of toxicity is generally lower for natural products compared to synthetic molecules. However, it is important to note that natural products have not undergone the same rigorous safety trials as FDA-approved drugs.

The figure below displays the condensed top 100 hit list of QFVinardo and QFVina methods for the 6LU7 target. All CSV files containing the hit lists for all three targets are provided in the supplementary material.

It is interesting to note that despite using the same QM conformations and performing identical docking runs, simply changing the scoring function for intermolecular interactions between the protein and the ligand results in only 23 out of approximately 60 molecules being the same in the condensed top 100 lists for QFVinardo and QFVina. It is also important to recognize that the estimated binding energy range for the entire condensed top 100 hit list is less than 1 kcal/mol for QFVinardo and just over 1 kcal/mol for QFVina. Given that the accuracy of empirical scoring functions for intermolecular interactions and desolvation energies is likely much worse than 1 kcal/mol, I recommend considering the entire list from both docking hit lists for further lead optimization steps.

Comparing the estimated binding energies of the QFVinardo hit list with those for FDA-approved drugs, one can observe that *Niclosamide*, a proven potent drug against COVID-19, ranks 4th on our FDA list with an estimated binding energy of −6.54 kcal/mol using the Vinardo intermolecular scoring function combined with DFT-D4 conformational strain energies. There are 19 natural compounds on the QFVinardo hit list with stronger estimated binding energies than −6.54 kcal/mol. Therefore, considering the entire QFVinardo and QFVina condensed top 100 hit lists, I believe there is a chance of finding non-toxic natural compounds that effectively inhibit the 6LU7 protein.

One example from the list is the molecule with COCONUT ID *CNP0415554*, a typical drug-like and drug-sized molecule, ranking 4th on the QFVinardo list and at the top of the QFVina list. The figure below illustrates the intermolecular interactions involving several hydrogen bonds with the surrounding residues from different parts of the molecule, suggesting potentially strong inhibition. The geometry of the given conformation of the molecule appears to accommodate these attractive interactions very well.

Visual presentations like these often highlight specific docking solutions, but my concern is that, despite their visual appeal, it is difficult to determine whether the conformation facilitating such hydrogen bond networks in the active site has 0.1 kcal/mol, 1 kcal/mol, 10 kcal/mol of conformational strain energy, or somewhere in between. This uncertainty regarding ligand conformation can often be a critical factor, especially when the estimated protein-ligand interaction energies across the entire hit list vary by only 1 or a few kcal/mol. However, in this case, we know that the conformation used is the lowest-energy QM conformation available for this molecule in our database. This knowledge eliminates at least one significant source of uncertainty in the docking results, highlighting the advantage that QFVina and QFVinardo docking methods offer.

## Conclusions and Prospects

I introduced the QFVina and QFVinardo schemes as two new docking methods within the AutoDock Vina family. Both methods perform rigid body ligand docking using the respective Vina or Vinardo scoring functions, leveraging low-energy QM-based conformations from our precomputed database. The overall scoring function is the sum of the docking scoring function and the high-quality DFT-D4 conformational strain energy, obtained from the QM conformational database and originally calculated by the QFConfsearchDFT application using the rev-SCAN functional and def2-TZVP basis set.

Our findings demonstrate that traditional flexible docking often produces highly strained conformer geometries. The final hit lists generated using low-energy, physically feasible QM conformers differ significantly from those obtained using traditional flexible ligand docking with the same intermolecular scoring function. The computational costs associated with these new methods have also been discussed in detail.

We demonstrated the results of the new methods using three protein targets from COVID-19 research and our QM conformational databases of FDA-approved drugs and natural products. While the focus of this paper is on the theoretical comparison between the new methods and traditional flexible ligand docking, a few existing drugs, such as (S)-3-Hydroxy-Midostaurin, and some natural compounds emerged as promising candidates for further investigation with higher-level computational methods and experimental validation for inhibiting key proteins involved in viral replication.

The true potential of the QFVina and QFVinardo methods, and similar approaches that utilize precomputed high-quality conformational libraries, warrants further exploration. Based on the results presented in this article, I believe that the QFVina and QFVinardo schemes provide enhanced accuracy and reliability for docking projects.

In light of these findings, I plan to pursue two main groups of projects:

1. Conduct preliminary docking studies for drug repurposing for critical, life-altering, or deadly diseases using the current FDA-approved drugs and natural product-based QM conformational libraries.
2. Expand the QM conformational database to include all approved drugs worldwide and significantly extend the natural product-based conformational library.

## Figures and Tables

**Fig1:**
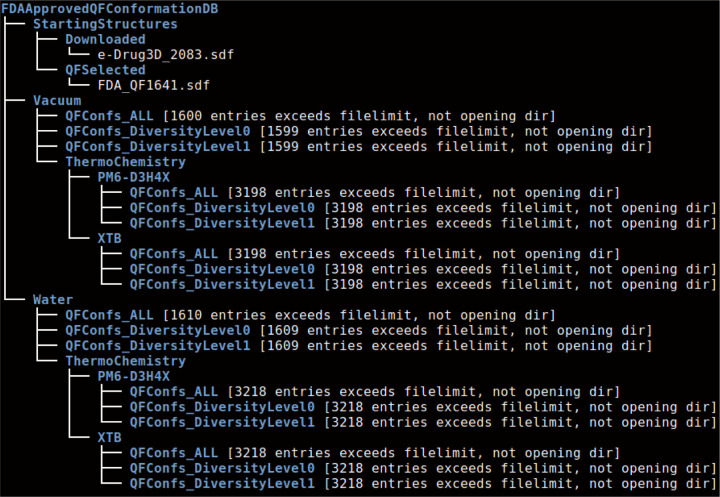
Directory structure and file counts of the current QM conformation database for FDA approved drugs

**Fig2:**
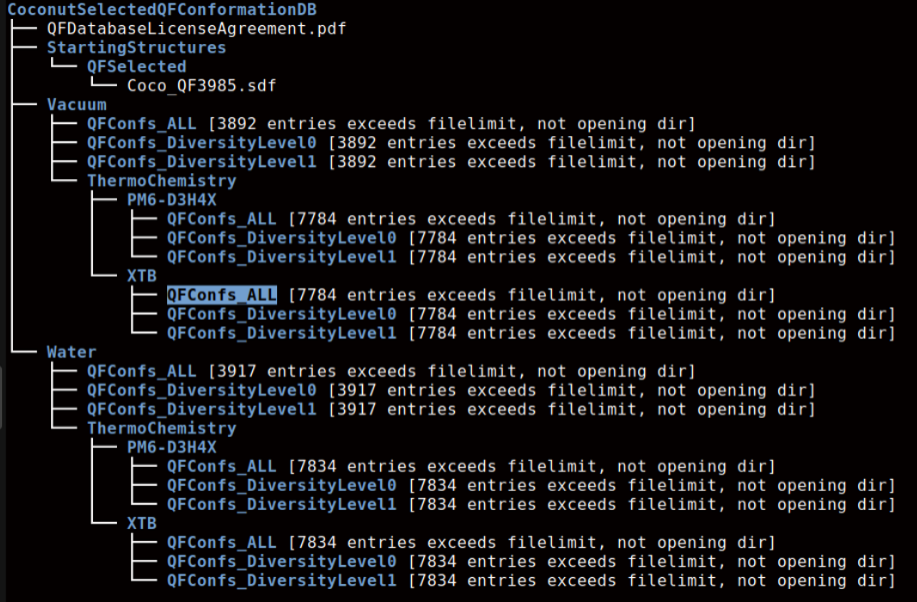
Directory structure and file counts of the current QM conformation database for the COCONUT natural product library

**Fig5:**
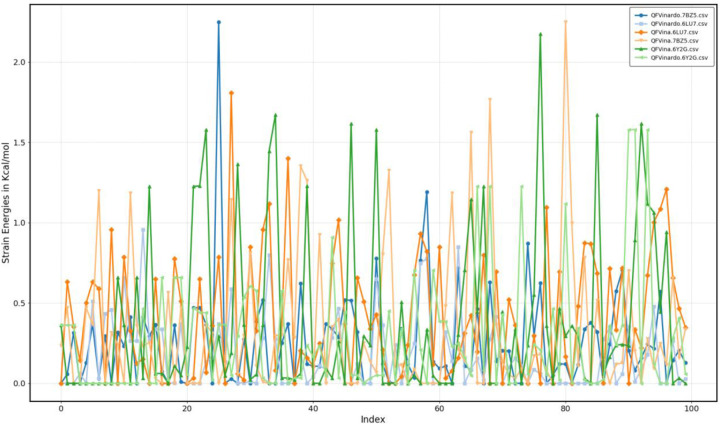
Conformational strain energies of the top100 hits in QFVina and QFVinardo dockings

**Fig6:**
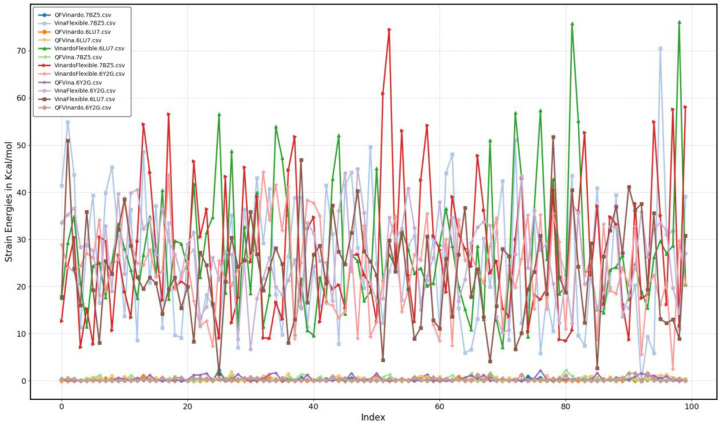
Strain energies of the top100 hits in flexible ligand docking using Vina and Vinardo scoring function

**Fig7:**
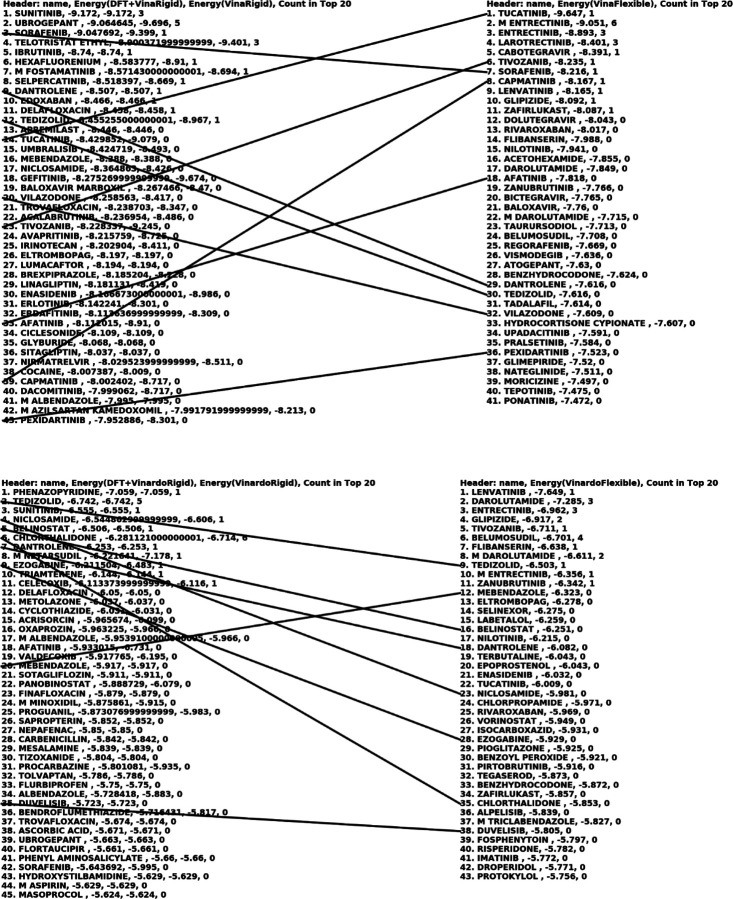
Top hit lists with QFVina and QFVinardo docking and traditional flexible ligand docking with Vina and Vinardo scoring functions for the 6LU7 target^a^ a: The lines are connecting the same drug molecules, and the last column everywhere shows how many times the same drug occurs in the top20 of the top100 hit list with different poses and conformations.

**Fig8:**
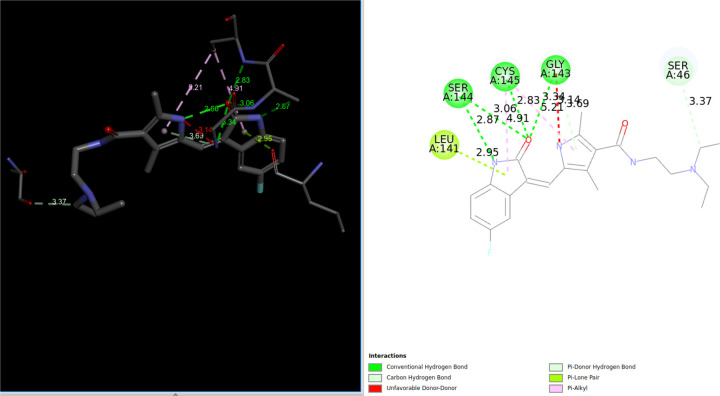
Intermolecular interactions between the lowest energy QM conformation of *Sunitinib* and the 6LU7 protein target

**Fig10:**
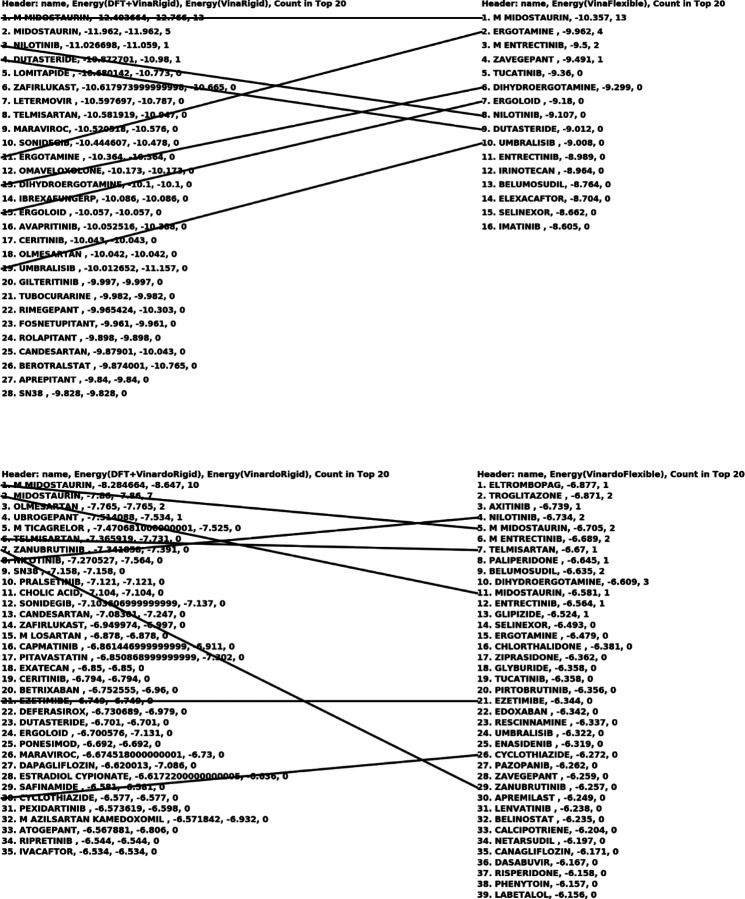
Top hit lists with QFVina and QFVinardo docking paired and compared with traditional flexible ligand docking with Vina and Vinardo scoring functions for the 6Y2G target^a^ a: The lines are connecting the same drug molecules, and the last column everywhere shows how many times the same drug occurs in the top20 of the top100 hit list with different poses and conformations.

**Fig11:**
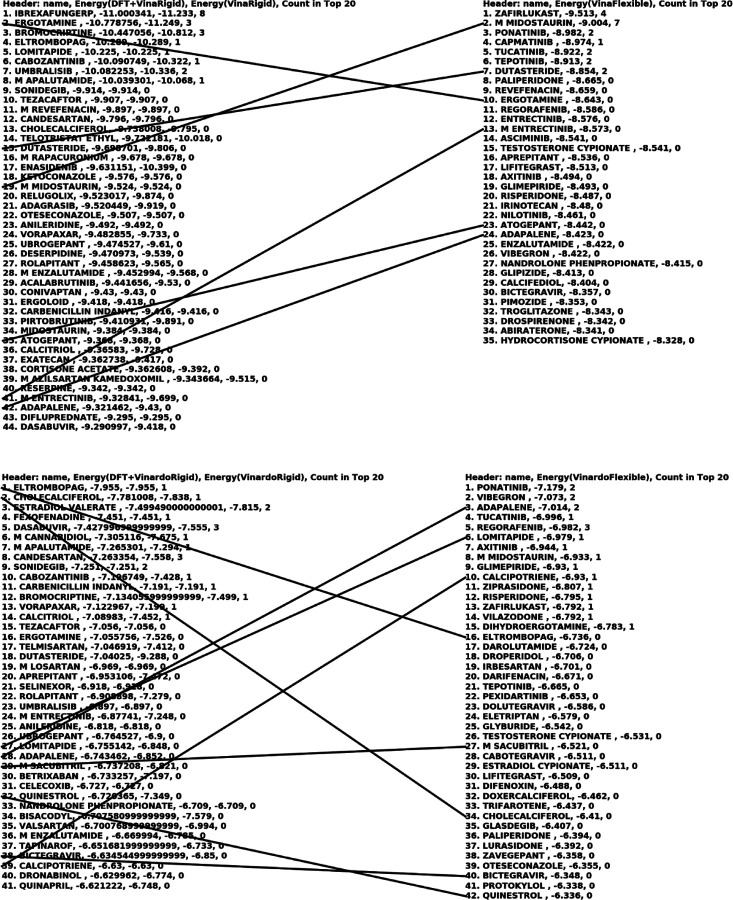
Top hit lists with QFVina and QFVinardo docking paired and compared with traditional flexible ligand docking with Vina and Vinardo scoring functions for the 7BZ5 target^a^ a: The lines are connecting the same drug molecules, and the last column everywhere shows how many times the same drug occurs in the top20 of the top100 hit list with different poses and conformations.

**Fig12:**
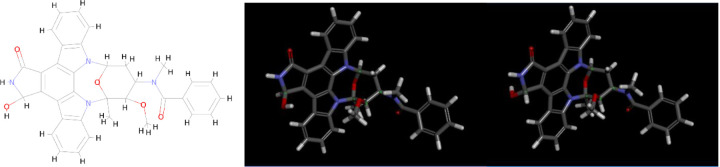
*3-Hydroxy Midostaurin* with 2D depiction and 3D geometries of both stereoisomers

**Fig13:**
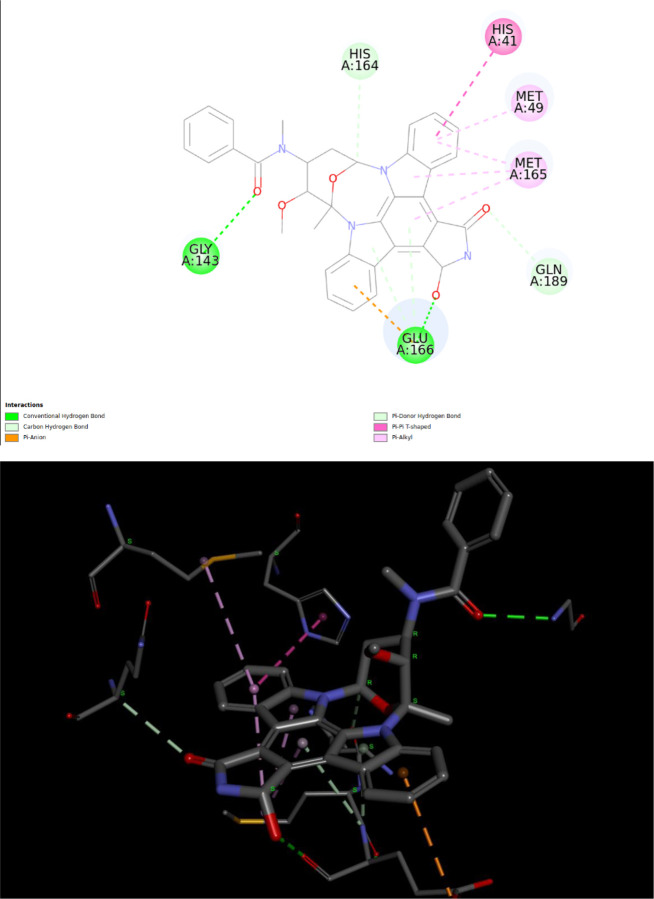
Visualizations of different intermolecular interactions in between *3-hydroxy Midostaurin* and the 6Y2G target protein using the pose from the hit lists of QFVinardo

**Fig14:**
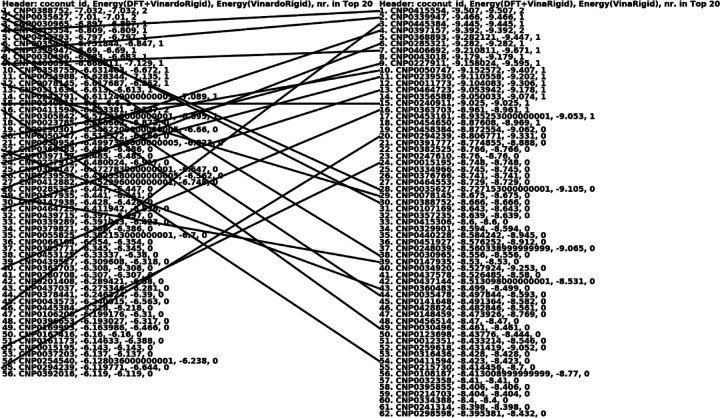
Top hit lists with QFVinardo and QFVina docking for the 6LU7 target. The lines are connecting the same drug molecules, and the last column shows how many times the same drug occurs in the top20 of the top100 hit list with different poses and conformations

**Fig15:**
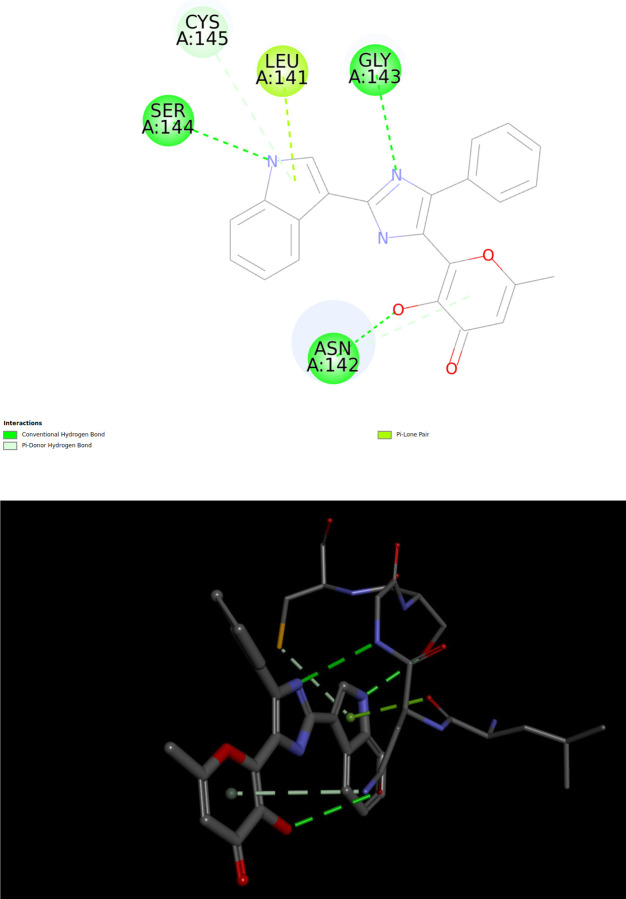
Visualizations of different intermolecular interactions in between CNP0415554 and the 6LU7 target protein using the pose from the hit lists of QFVinardo

**Table1:** Statistics for conformational relative energies and conformational ranking errors for a set of 2954 geometries of small molecules. All energy values are in kcal/mol. Entries in bold are new data from this work. All DFT calculations in bold have been performed with the 2024 version of the QFDFT program[22]

Method versus dlpno	Mean of RMSDs of Relative Energies	Median of RMSDs of Relative Energies	Mean of Ranking Errors	Median of Ranking Errors
mp2	0.253	0.145	0.348	0
b973c	0.361	0.269	0.859	0
Pbe	0.423	0.259	1.020	0
pbeSVP	0.486	0.329	1.223	0
pbeh3c	0.423	0.259	1.020	0
b3lypTZ	0.287	0.229	0.767	0
b3lypSVP	0.423	0.292	1.026	0
**DFT-D4(revScan def2-TZVP)**	**0.364**	**0.271**	**0.951**	**0**
**DFT-D4(R2Scan def2-TZVP)**	**0.413**	**0.269**	**0.941**	**0**
DFT-D4(revScan 6–311G**)	**0.482**	**0.285**	**1.105**	**0**
DFT-D4(R2Scan 6–311G**)	**0.469**	**0.283**	**1.056**	**0**
**DFT-D4(revTPSS def2-TZVP)**	**0.324**	**0.247**	**0.852**	**0**
DFT-D4(revTPSS 6–311G**)	**0.389**	**0.265**	**0.934**	**0**
DFT(revM06_L 6–311G**)	**0.430**	**0.298**	**0.895**	**0**
gfn0	1.083	0.587	2.561	2
gfn1	0.848	0.455	1.807	1
gfn2	0.784	0.470	1.649	1
pm7E	1.277	0.759	4.538	5
pm7HOF	1.852	0.824	5.023	5
Mmff	8.685	0.795	2.882	2
Uff	1228.754	4.395	3.170	2
Gaff	880.814	1.299	2.793	2
**GFN-FF**[23]	**1.300**	**0.593**	**2.836**	**2**
ani1x	0.993	0.583	2.059	1
ani1cc	0.899	0.583	1.846	1
ani2	0.971	0.616	2.115	1
**GAFF Redo with obabel**	**9.562**	**5.749**	**2.731**	**2**
**MMFF Redo with obabel**	**1.808**	**0.788**	**2.836**	**2**
**MMFF94s with obabel**	**1.871**	**0.813**	**2.967**	**2**
**Ghemical with obabel**	**25.448**	**6.639**	**2.872**	**2**
**UFF Redo with obabel**	**43.897**	**17.889**	**3.134**	**2**
**Open-FF (Sage)**[24]	**19.505**	**0.762**	**2.095**	**1**
**Open-FF (Parsley)**[25]	**20.449**	**0.791**	**2.282**	**1**

**Table2:** Statistics for conformational relative energies and conformational ranking errors for a set of 6612 geometries of small molecules. All energy values are in kcal/mol. Entries in bold are new data from this work. All DFT calculations in bold have been performed with the 2024 version of the QFDFT program[22]

Method versus dlpno	Mean of RMSDs of Relative Energies	Median of RMSDs of Relative Energies	Mean of Ranking Errors	Median of Ranking Errors
mp2	0.282	0.150	0.361	0
b973c	0.385	0.271	0.795	0
Pbe	0.450	0.274	0.914	0
pbeSVP	0.574	0.349	1.102	0
pbeh3c	0.450	0.274	0.914	0
b3lypTZ	0.312	0.227	0.713	0
b3lypSVP	0.516	0.306	0.967	0
**DFT-D4(revScan def2-TZVP)**	**0.392**	**0.279**	**0.895**	**0**
**DFT-D4(R2Scan def2-TZVP)**	**0.436**	**0.283**	**0.878**	**0**
DFT-D4(revScan 6–311G**)	**0.496**	**0.299**	**1.016**	**0**
DFT-D4(R2Scan 6–311G**)	**0.484**	**0.292**	**0.955**	**0**
**DFT-D4(revTPSS def2-TZVP)**	**0.373**	**0.268**	**0.809**	**0**
DFT-D4(revTPSS 6–311G**)	**0.433**	**0.290**	**0.889**	**0**
DFT(revM06_L 6–311G**)	**0.450**	**0.315**	**0.843**	**0**
gfn0	1.194	0.650	2.489	1
gfn1	0.935	0.525	1.801	1
gfn2	0.920	0.522	1.785	1
pm7E	1.493	0.820	4.448	4
pm7HOF	2.094	0.921	4.901	5
Mmff	18.146	0.905	2.676	2
Uff	1156.557	4.848	3.350	3
Gaff	858.227	1.390	2.773	2
**GFN-FF**[23]	**1.372**	**0.685**	**2.840**	**2**
**GAFF Redo with obabel**	**9.777**	**5.852**	**2.687**	**2**
**MMFF Redo with obabel**	**1.737**	**0.839**	**2.638**	**2**
**MMFF94s with obabel**	**1.791**	**0.873**	**2.732**	**2**
**Ghemical with obabel**	**25.299**	**7.211**	**3.169**	**3**
**UFF Redo with obabel**	**42.720**	**17.887**	**3.332**	**3**
**Open-FF (Sage)**[24]	**21.391**	**0.806**	**2.210**	**1**
**Open-FF (Parsley)**[25]	**20.911**	**0.846**	**2.312**	**1**

**Table3:** Intervals for protein-ligand binding energies for the top 100 docking results by using rigid protein and rigid ligand docking with QFVina and QFVinardo scoring function by considering all conformations from the current QF FDA Drug Repurposing Conformational Database. All values are in kcal/mol

Scoring function	QFVina			QFVinardo		
Target	6LU7	6Y2G	7BZ5	6LU7	6Y2G	7BZ5
Strongest Binding Energy	−9.17	−12.40	−11.00	−7.06	−8.28	−7.96
100^th^ Strongest Binding Energy	−7.95	−9.83	−9.26	−5.62	−6.51	−6.62

**Table4:** As in Table3, but for Vina and Vinardo instead of QFVina and QFVinardo respectively

Scoring function	Vina			Vinardo		
Target	6LU7	6Y2G	7BZ5	6LU7	6Y2G	7BZ5
Strongest Binding Energy	−9.65	−10.36	−9.51	−7.65	−6.88	−7.18
100^th^ Strongest Binding Energy	−7.47	−8.60	−8.32	−5.76	−6.16	−6.34

**Table5:** Statistics of the conformational strain energies of the top100 hits in QFVina and QFVinardo dockings. All values are in kcal/mol

Scoring function	QFVina			QFVinardo		
Target	6LU7	6Y2G	7BZ5	6LU7	6Y2G	7BZ5
Minimum	0.00	0.00	0.00	0.00	0.0	0.0
Maximum	1.81	2.18	2.25	0.96	1.58	2.25
Mean	0.41	0.38	0.33	0.17	0.28	0.25
Median	0.34	0.14	0.15	0.07	0.10	0.15
Standard deviation	0.39	0.53	0.44	0.23	0.38	0.31
Mean Absolute Deviation	0.32	0.40	0.32	0.18	0.28	0.21

**Table6:** Statistics of the strain energies of the top100 hits in flexible ligand docking using Vina and Vinardo scoring function. All values are in kcal/mol

Scoring function	Vina			Vinardo		
Target	6LU7	6Y2G	7BZ5	6LU7	6Y2G	7BZ5
Minimum	1.45	6.677	−0.12	7.17	2.55	7.13
Maximum	51.69	44.97	70.53	76.12	44.26	74.54
Mean	23.23	26.21	25.36	27.59	23.99	27.42
Median	23.45	26.49	24.60	24.27	24.48	23.77
Standard deviation	10.24	8.61	13.97	13.70	9.23	14.85
Mean Absolute Deviation	7.99	7.09	11.78	10.16	7.31	12.12
